# Required Elements in tRNA for Methylation by the Eukaryotic tRNA (Guanine-*N*^2^-) Methyltransferase (Trm11-Trm112 Complex)

**DOI:** 10.3390/ijms23074046

**Published:** 2022-04-06

**Authors:** Yu Nishida, Shiho Ohmori, Risa Kakizono, Kunpei Kawai, Miyu Namba, Kazuki Okada, Ryota Yamagami, Akira Hirata, Hiroyuki Hori

**Affiliations:** Department of Materials Science and Biotechnology, Graduate School of Science and Engineering, Ehime University, 3 Bunkyo-cho, Matsuyama 790-8577, Japan; yu_nishida_ehime_u@yahoo.co.jp (Y.N.); sh10_2790@yahoo.co.jp (S.O.); risa.kakizono@gmail.com (R.K.); kumpei0405@gmail.com (K.K.); miyu.n.014744687@gmail.com (M.N.); aporon8okada2006@yahoo.co.jp (K.O.); yamagami.ryota.bn@ehime-u.ac.jp (R.Y.); ahirata@tokushima-u.ac.jp (A.H.)

**Keywords:** RNA modification, tRNA methyltransferase, modified nucleoside

## Abstract

The *Saccharomyces cerevisiae* Trm11 and Trm112 complex (Trm11-Trm112) methylates the 2-amino group of guanosine at position 10 in tRNA and forms *N*^2^-methylguanosine. To determine the elements required in tRNA for methylation by Trm11-Trm112, we prepared 60 tRNA transcript variants and tested them for methylation by Trm11-Trm112. The results show that the precursor tRNA is not a substrate for Trm11-Trm112. Furthermore, the CCA terminus is essential for methylation by Trm11-Trm112, and Trm11-Trm112 also only methylates tRNAs with a regular-size variable region. In addition, the G10-C25 base pair is required for methylation by Trm11-Trm112. The data also demonstrated that Trm11-Trm112 recognizes the anticodon-loop and that U38 in tRNA^Ala^ acts negatively in terms of methylation. Likewise, the U32-A38 base pair in tRNA^Cys^ negatively affects methylation. The only exception in our in vitro study was tRNA^Val^_AAC1_. Our experiments showed that the tRNA^Val^_AAC1_ transcript was slowly methylated by Trm11-Trm112. However, position 10 in this tRNA was reported to be unmodified G. We purified tRNA^Val^_AAC1_ from wild-type and *trm11* gene deletion strains and confirmed that a portion of tRNA^Val^_AAC1_ is methylated by Trm11-Trm112 in *S. cerevisiae*. Thus, our study explains the m^2^G10 modification pattern of all *S. cerevisiae* class I tRNAs and elucidates the Trm11-Trm112 binding sites.

## 1. Introduction

**Figure 1:**
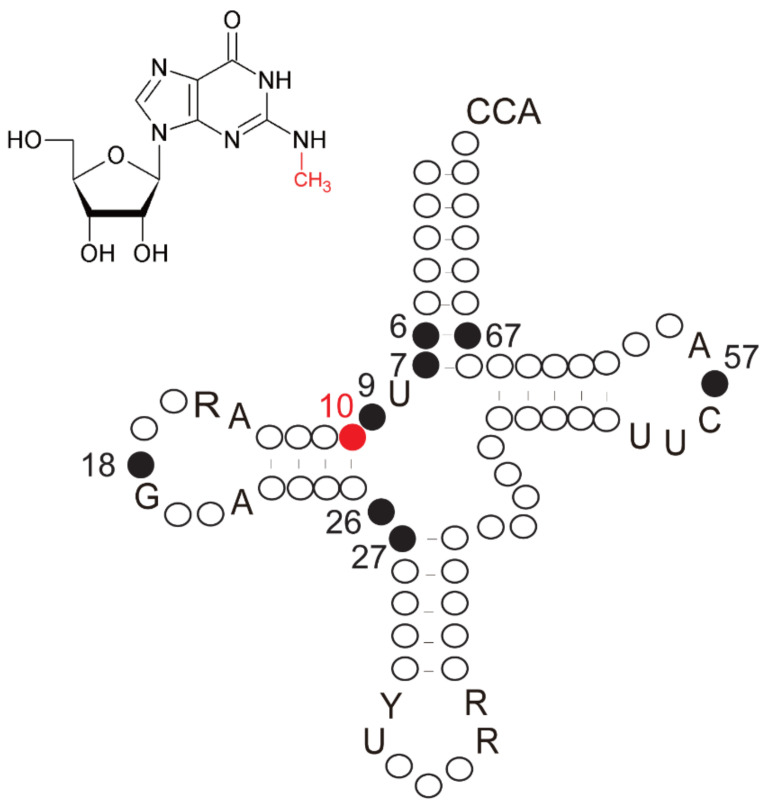
*N*^2^-methylguanosine and its positions in tRNA. The structure of m^2^G is shown in the upper left. The methyl group of m^2^G is highlighted in red. This methyl group is introduced by site-specific tRNA (guanine-*N*^2^-) methyltransferase using S-adenosyl-L-methionine as a methyl group donor. The m^2^G modification has been found at positions 6, 7, 9, 10, 18, 26, 27, 57 and 67 in tRNAs: these positions are filled in the cloverleaf structure of tRNA. The numbers show the positions in tRNA. Conserved residues in tRNA are shown by letters: R, purine; Y, pyrimidine. The Trm11-Trm112 complex methylates G at position 10 (marked in red) of tRNA.

To date, more than 100 modified nucleosides have been found in tRNA [1], with the most frequent modifications being methylation of bases and/or the 2′-OH of riboses [2,3]. Of the methylated nucleosides, *N*^2^-methylguanosine (m^2^G) has been found at positions 6, 7, 9, 10, 18, 26, 27, 57 and 67 in tRNAs from eukaryotes, archaea and eubacteria (Figure 1). These modifications are post-transcriptionally introduced by site-specific tRNA (guanine-*N*^2^-) methyltransferases (EC 2.1.1.32).

The m^2^G modification at position 10 (m^2^G10) is often observed in tRNAs from archaea and eukaryotes. In *Saccharomyces cerevisiae*, m^2^G10 is synthesized by a complex of two proteins, namely Trm11 and Trm112 [4]. Trm11 works as a catalytic subunit while Trm112 is a regulatory subunit [4,5,6,7]. Although the crystal structure of the Trm11-Trm112 complex from *S. cerevisiae* has not been reported, a structural model has been proposed based on the structures of archaeal orthologs and a combination of biochemical, biophysical and bioinformatic studies [8,9,10]. Trm11 is a typical Rossmann fold S-adenosyl-L-methionine (AdoMet)-dependent methyltransferase (COG 1041) with a thiouridine synthase, methyltransferase and pseudouridine synthase (THUMP) domain fused to its N-terminal region [4,5,8,9,10]. The THUMP domain is frequently observed in tRNA modification enzymes [11,12,13,14,15,16,17,18,19] and is often required for binding to the CCA terminal region of tRNA [8,9,10,19,20]. In the Trm11-Trm112 complex, Trm112 activates the tRNA methyltransferase activity of Trm11 [4,9]. It should be mentioned that Trm112 activates not only Trm11 but also other methyltransferases in yeast (for example, Trm9 [21], Bud23 [22,23] and Mtq2 [6,24,25,26]). Furthermore, a human ortholog of Trm112, TRMT112, interacts with at least seven human methyltransferases (WBSCR22 (responsible for the formation of 7-methylguanosine at position 1636 in 18S rRNA) [27], METTL5 (responsible for the formation of *N*^6^-methyladenosine at position 1832 in 18S rRNA) [28], HEMK2 (responsible for the methylation of a glutamine side chain of eRF1 protein) [29], ALKBH8 (responsible for 5-methoxycarbonylmethyluridine derivatives at position 34 in tRNA) [30,31,32,33], TRMT11 (probably responsible for m^2^G10 in tRNA, although the enzymatic activity has not yet been confirmed experimentally) [4,34], THUMPD2 (function unknown) [34] and THUMPD3 (responsible for the production of m^2^G6 and m^2^G7 in tRNA)) [19]. Thus, in eukaryotes, Trm112 acts as a hub-protein, which regulates tRNA, rRNA and protein methylation [6,34]. In archaea, two types of m^2^G10 modification systems have been reported. *Archaeoglobus fulgidus* [10] and *Halloferax volcanii* [35] Trm11 require an archaeal ortholog of Trm112 for enzymatic activity. In contrast, *Pyrococcus abyssi* [5] and *Thermococcus kodakarensis* [8] Trm11 proteins do not require Trm112 for enzymatic activity. Furthermore, in the case of archaeal tRNA, m^2^G10 is further methylated to *N*^2^, *N*^2^-dimethylguanosine at position 10 (m^2^_2_G10) via a second methylation by the archaeal Trm11-Trm112 complex [10] or archaeal Trm11 alone [5,8].

The methyl group of m^2^G10 does not disturb the formation of a Watson–Crick base pair with C25 in the L-shaped tRNA structure [36]. The hydrophobic effect of the methyl group of m^2^G10 probably stabilizes the D-stem structure. The growth rate of a *S. cerevisiae trm11* gene deletion strain is comparable with that of the wild-type strain under laboratory conditions [4]. However, a *trm1*-*trm11* double gene deletion strain shows obvious growth defect [4]. Because Trm1 is the tRNA methyltransferase responsible for the formation of m^2^_2_G26 [37,38], the study in [4] strongly suggested that the m^2^G10 modification works in coordination with other modification(s) in tRNA. In the case of archaea, m^2^_2_G10 is suggested to prevent incorrect folding of tRNA [39], and the *trm11* gene deletion strain of *T. kodakarensis* shows severe growth retardation at high temperatures (95 °C) [40,41].

**Figure 2:**
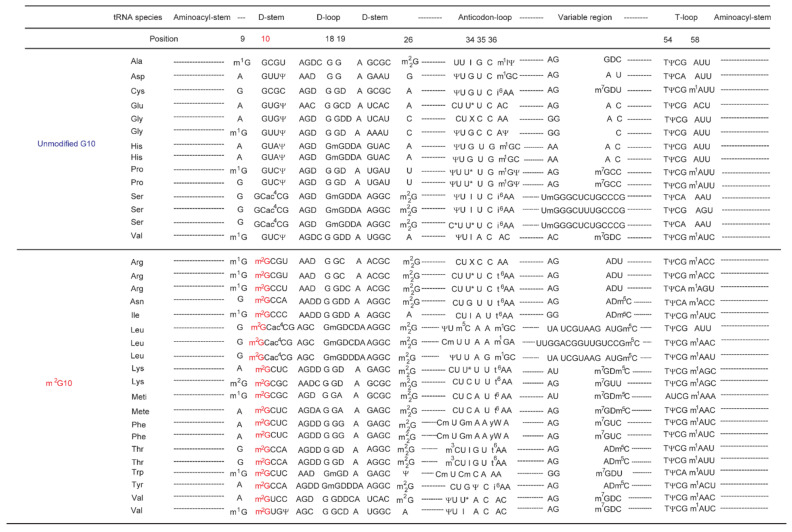
Comparison of *S. cerevisiae* tRNA sequences. The sequences of D-arms, anticodon-loops, variable regions and T-loops in *S. cerevisiae* tRNAs are compared. The abbreviations are as follows; m^1^G, 1-methylguanosine; D, dihydrouridine; I, inosine; m^1^I, 1-methylinosine; T, thymidine; Ψ, pseudouridine; i^6^A, *N*^6^-isopetenyladenosine; m^7^G, 7-methylguanosine; m^1^A, 1-methyladenosine; U*, U-modification; X, unidentified modification; Gm, 2′-*O*-methylguanosine; Um, 2′-*O*-methyluridine; ac^4^C, 4-acetylcytidine; m^5^C, 5-methylcytidine; m^3^C, 3-methylcytidine; t^6^A, *N*^6^-threonylcarbamoyladenosine; and yW, wyosine. The position of a modification caused by Trm11-Trm112 (m^2^G10) is highlighted in red. The sequences in the aminoacyl-stem, anticodon-stem and T-stem are not shown.

The studies of m^2^G10 in tRNA and Trm11 described above have been reported over the past two decades. However, one important question remains: why is the m^2^G modification only observed in a limited number of tRNA species in *S. cerevisiae* (Figure 2 and References [42,43]). In this paper, the numbering of position in tRNA is according to Reference [44]. As shown in Figure 2, the recognition site(s) of *S. cerevisiae* Trm11-Trm112 in tRNA cannot simply be predicted from the sequences of modified and unmodified tRNAs. Fortunately, during the course of this study, a structural model of the Trm11-Trm112 complex was proposed [9]. In the current study, we performed biochemical studies to clarify the required elements in tRNA for methylation by the Trm11-Trm112 complex and discuss the experimental results based on the proposed structural model.

## 2. Results

### 2.1. Co-Expression of Trm11 and Trm112 in Escherichia coli Cells and Purification

In our previous study, we reported the production of a Trm11-Trm112 complex in a wheat germ cell-free expression system to investigate whether the cell-free system can synthesize a multiple protein complex [45]. In the current study, we constructed a co-expression system for Trm11-Trm112 in *Escherichia coli* cells and purified Trm11-Trm112 (Supplementary Figure S1). The details are described in Supplementary File S1.

### 2.2. Measurement of Methyltransferase Activity of Trm11-Trm112 Complex

We measured the methyltransferase activity of purified Trm11-Trm112 in several conditions and noticed that the diluted enzyme rapidly decreased in activity (Supplementary Figure S2). During the course of this study, a time course assay of Trm11-Trm112 activity toward a tRNA^Ile^_AAU_ transcript was reported and the methyl-transfer activity of this Trm11-Trm112 was also observed to decrease rapidly [9]. Thus, this phenomenon is not caused by tRNA species but is probably caused by the dissociation of Trm11-Trm112 subunits in the reaction mixture. When 0.10 µM Trm11-Trm112, 10.0 µM yeast tRNA^Phe^ transcript and 20.1 µM AdoMet (20 µM non-radioisotope labeled AdoMet and 0.06 µM ^3^H-AdoMet) were incubated at 30 °C, ^3^H-methyl group incorporation into the tRNA^Phe^ transcript showed linearity for the initial 2 min (Supplementary Figure S2). Therefore, in this study, we measured the initial velocities of the methyl-transfer reaction for 2 min periods in the above conditions. Furthermore, it should be mentioned that Trm11-Trm112 strictly recognizes the CCA terminal region of tRNA, as described later in this paper. Therefore, we prepared tRNA transcript using 2′-*O*-methylated DNA for the construction of a template to avoid adding extra nucleotide(s) to the CCA terminal region. To visualize the methyl group incorporation, ^14^C-AdoMet was used. Details are described in the Materials and Methods section.

### 2.3. Precursor tRNA^Phe^ Is Not Methylated by the Trm11-Trm112 Complex

**Figure 3:**
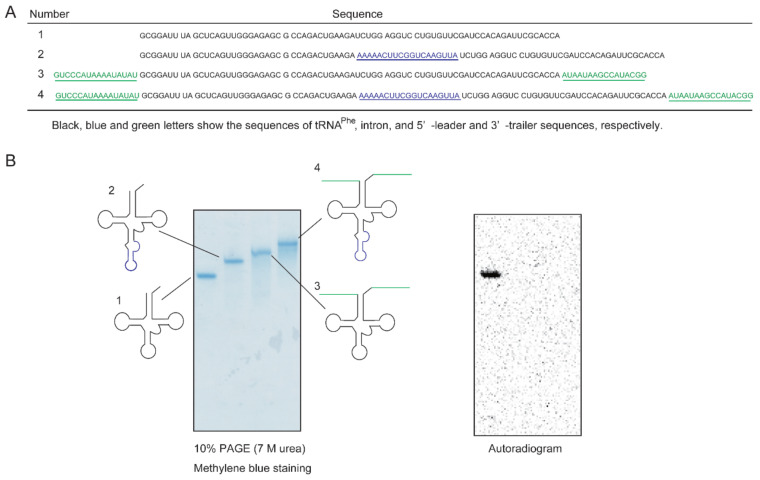
Precursor tRNA^Phe^ transcript is not methylated by the Trm11-Trm112 complex. (**A**) Sequences of precursor tRNA^Phe^ transcript. Transcript 1 is the mature-size tRNA^Phe^ transcript. Blue and green letters show the sequences of intron, and 5′-leader and 3′-trailer regions. (**B**) ^14^C-methylated transcripts were separated by 10% PAGE (7 M urea) (**left**). An autoradiogram of the gel was obtained (**right**).

As described in the Introduction, the substrate tRNA recognition mechanism of Trm11-Trm112 cannot simply be predicted by the sequences of tRNAs from *S. cerevisiae*. In *S. cerevisiae*, an intron in precursor tRNA is removed in the cytoplasm [46,47] and precursor tRNA is repeatedly transported between cytoplasm and nucleus [46,48,49]. It should be mentioned that Trm11 is localized in the cytoplasm [4]. Furthermore, although the 5′-cap structure is attached to four precursor tRNAs (tRNA^Ile^_UAU_, tRNA^Leu^_CAA_, tRNA^Lys^_UUU_ and tRNA^Trp^_CCA_) during the transport process, the attachment of the cap structure to precursor tRNA^Phe^ has not been reported [49]. At the beginning of this study, we assumed that intron, and/or leader and trailer sequences of precursor tRNAs might have an effect on the substrate tRNA recognition by Trm11-Trm112. To address this issue, we prepared four types of tRNA^Phe^ transcripts (Figure 3A). Transcript 1 is the mature-size tRNA^Phe^ transcript. Transcript 2 has an intron at the canonical position between positions 38 and 39. Transcript 3 has 5′-leader and 3′-trailer sequences.

Transcript 4 has an intron, and 5′-leader and 3′-trailer sequences. These transcripts were incubated at 30 °C for 2 min with Trm11-Trm112 and ^14^C-AdoMet, and then, RNAs were recovered from phenol-chloroform treatment. The RNA samples were loaded onto a 10% polyacrylamide gel containing 7 M urea (PAGE (7 M urea)), and an autoradiogram of the gel obtained (Figure 3B).

As shown in Figure 3B, the ^14^C-methyl group was incorporated only into the mature-size tRNA^Phe^ transcript. Thus, precursor tRNAs are not substrates for Trm11-Trm112. The results suggest that the m^2^G10 modification by Trm11-Trm112 is one of the final events in tRNA maturation in *S. cerevisiae* cells. Furthermore, because the presence of an intron affects methylation by Trm11-Trm112, one of the recognition sites of Trm11-Trm112 is predicted to be the anticodon-loop. Moreover, because the 5′-leader and 3′-trailer sequences negatively affects methylation, Trm11-Trm112 is predicted to recognize the end(s) of the aminoacyl-stem in addition to the anticodon-loop of tRNA.

### 2.4. Deletion and Stem-Disruptant Mutants of tRNA^Phe^ Transcripts Are Not Methylated by Trm11-Trm112

**Figure 4:**
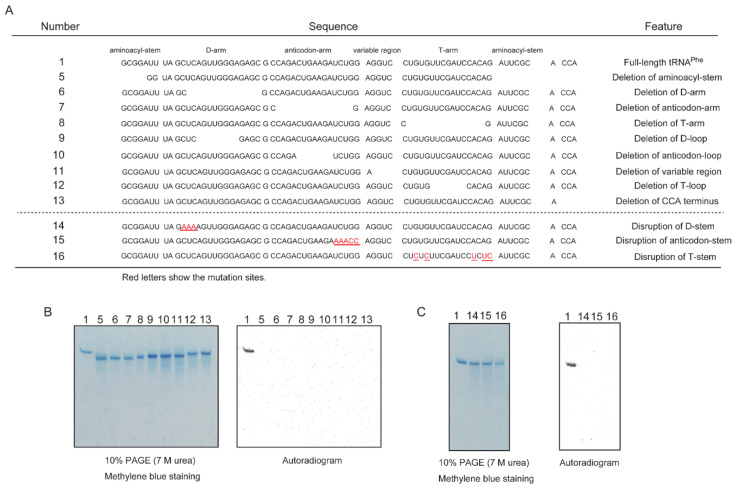
None of the deletion and stem-disruptant mutant tRNA^Phe^ transcripts are methylated by the Trm11-Trm112 complex. (**A**) Sequences of tRNA^Phe^ deletion mutants (Numbers 1–13) and stem-disruptant mutant (Numbers 14–16) are shown. (**B**) The deletion mutant tRNA^Phe^ transcripts were incubated with Trm11-Trm112 and ^14^C-AdoMet and then loaded onto a 10% polyacrylamide gel containing 7 M urea. After the electrophoresis, the gel was stained with methylene blue and dried (**left**). An autoradiogram of the gel was obtained (**right**). One full-length tRNA^Phe^ transcript was methylated by Trm11-Trm112. (**C**) Methylation of the stem-disruptant mutant tRNA^Phe^ transcripts was analyzed using the same method described in panel (**B**). Disruption of D-stem, anticodon-stem or T-stem abolishes methylation by Trm11-Trm112.

To clarify the recognition site(s) of Trm11-Trm112, we prepared twelve mutant tRNA^Phe^ transcripts (Figure 4A). Nine transcripts (transcripts 5–13) were deletion mutants and three transcripts (transcripts 14–16) were disruptant mutants of the stems. To our surprise, none of mutants were methylated by Trm11-Trm112 (Figure 4B,C). The experiments using precursor tRNA transcripts (Figure 3) suggest that the anticodon-loop and aminoacyl-stem are the recognition sites of the Trm11-Trm112 complex. However, with transcripts 6, 8, 9, 11, 14 and 16, their anticodon-arm and aminoacyl-stem are not mutated. These results suggest that the recognition sites of the Trm11-Trm112 complex are plentiful and are dispersed through the L-shaped tRNA structure.

### 2.5. The CCA Terminus Is Essential for Methylation by Trm11-Trm112

**Figure 5:**
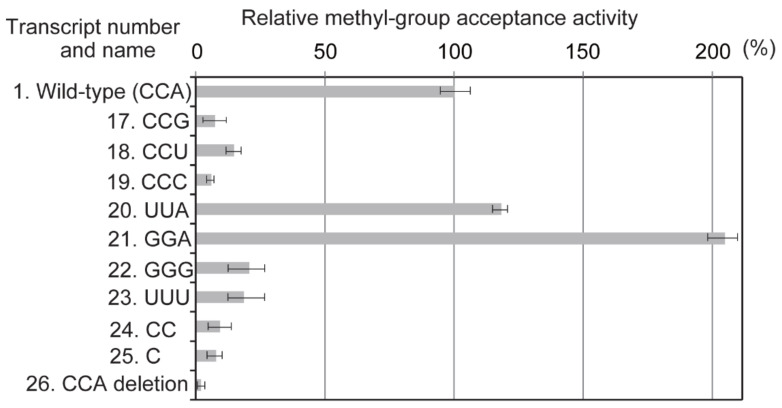
The CCA terminal region is essential for methylation by Trm11-Trm112. The CCA sequence of the wild-type tRNA^Phe^ transcript was substituted by other sequences. For example, transcript 17 possesses CCG instead of CCA. Transcripts 24, 25 and 26 are the deletion mutants of the CCA sequence. The initial velocity of methyl-transfer reaction towards the wild-type tRNA^Phe^ transcript is expressed as 100%. The data in this figure are averages of results from six independent experiments.

During the course of this study, we solved the crystal structure of *T. kodakarensis* Trm11 [8], which catalyzes the formation of m^2^G10 and m^2^_2_G10 in tRNA. *Thermococcus kodakarensis* Trm11 does not require a partner subunit (Trm112) for activity [8]. In the study, we reported that the THUMP domain in *T. kodakarensis* Trm11 recognizes the CCA terminus in tRNA [8]. The recognition of the 3′-end of substrate tRNA by a THUMP domain has also been reported for other tRNA modification enzymes [8,9,10,19,20]. For example, the crystal structure of complex of *Thermotoga maritima* ThiI (tRNA 4-thiouridine synthetase) and truncated tRNA revealed that the THUMP domain in ThiI captures the 3′-terminal region in tRNA [20]. Because eukaryotic Trm11 also possesses a THUMP domain [4], we considered that the 3′-terminal region of tRNA may be one of the recognition sites of Trm11-Trm112. To examine this idea, we prepared ten mutant tRNA^Phe^ transcripts, in which the CCA sequence was mutated or deleted (Figure 5).

When A76 was replaced by G, U or C (transcripts 17, 18 or 19), the methyl group acceptance activity was considerably decreased. These results reveal that the adenine base at position 76 is important for methylation by Trm11-Trm112. In contrast, cytosine bases at positions 74 and 75 are not important because mutant transcripts 20 and 21 were well methylated by Trm11-Trm112. However, the presence of nucleotides at positions 74 and 75 themselves is required for efficient methylation by Trm11-Trm112 because the methyl group acceptance activities of deletion mutants (transcripts 24, 25 and 26) were considerably decreased. These results show the importance of A76 and the ribose-phosphate backbone of the CCA terminus for recognition by Trm11-Trm112. During the course of this study, the methyl-transfer activity of Trm11-Trm112 towards a CCA deletion mutant of tRNA^Ile^ transcript was reported [9]. These results also highlight the importance of the CCA terminus for methylation by Trm11-Trm112 [9].

### 2.6. Requirement of the G10-C25 Base Pair and Interaction between the D-Arm and T-Arm

**Figure 6:**
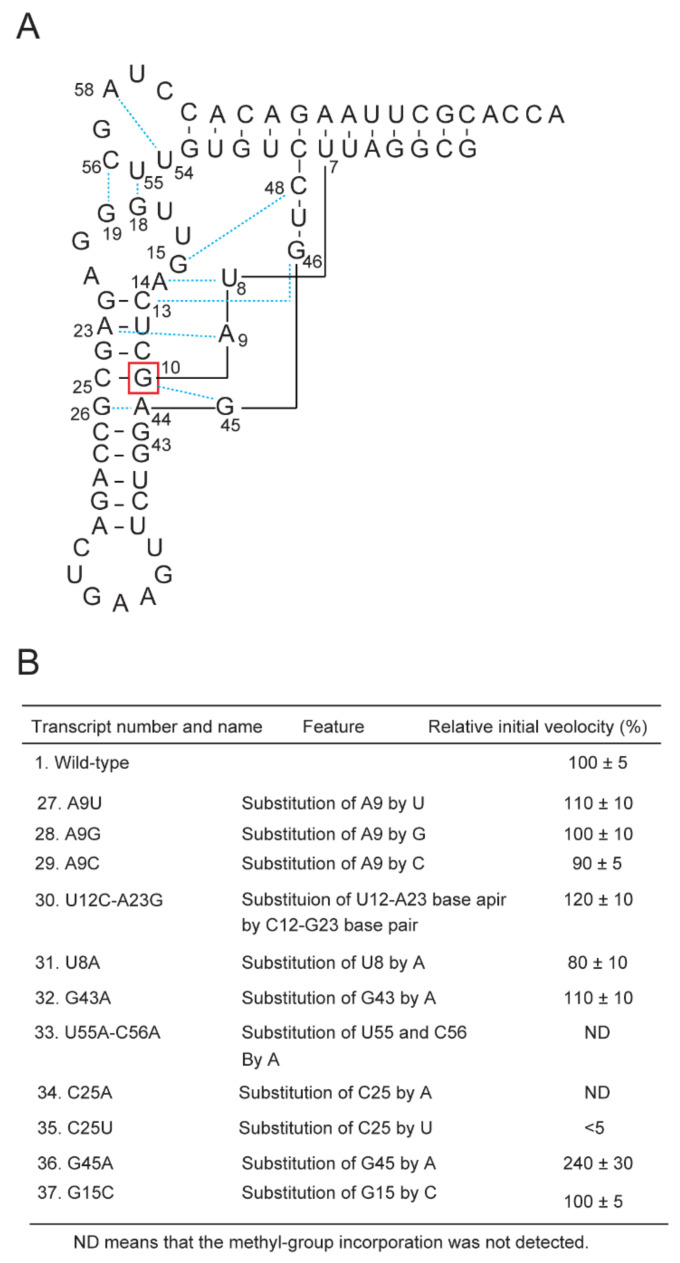
Effect of disruption of tertiary base pair or stem structure on methylation by Trm11-Trm112. (**A**) Tertiary base pairs (cyan dotted lines) are formed in tRNA^Phe^. The methylation site G10 is highlighted in a red square. The numbers show the positions in tRNA. (**B**) To disrupt tertiary base pairs, mutations were introduced into the tRNA^Phe^ transcript. For example, in the case of transcript 27, A9 in the tRNA^Phe^ transcript was substituted by U. As a result, the A9-A23 tertiary base pair was disrupted. The initial velocity of methyl-transfer to the wild-type tRNA^Phe^ transcript is expressed as 100%. The data in this figure are averages of the results from four independent experiments. “ND” means that methyl-transfer was not detected.

In the L-shaped tRNA^Phe^ structure, several tertiary base pairs are formed (blue dotted lines in Figure 6A and Reference [36]). We considered whether tertiary base pairs might be involved in the efficiency of methylation by Trm11-Trm112 because several tertiary base pairs are formed around the methylation site G10. We prepared eleven tRNA^Phe^ transcript variants and measured their methyl group acceptance activities (Figure 6B). Contrary to our expectation, the tertiary base pairs around G10 are not essential. However, when U55 and C56 were substituted by A55 and A56, the methyl-group acceptance activity was lost (transcript 33). This result shows that the interaction between the D-arm and T-arm is essential for methylation by Trm11-Trm112. Furthermore, when C25 was substituted by A or U (transcripts 34 and 35), the methyl group acceptance activity was nearly completely lost. Thus, these results reveal that the G10-C25 base pair is essential for the methylation by Trm11-Trm112. These results explain a part of the G10 methylation patterns presented in Figure 2. All tRNAs that possess U25 (tRNA^Asp^, two tRNA^Gly^ and two tRNA^Pro^) are not methylated by Trm11-Trm112 in vivo. Thus, U25 acts as a negative element for methylation by Trm11-Trm112. Furthermore, when the G10-G45 tertiary base pair was disrupted, the methyl-group acceptance activity was clearly increased (transcript 36). This result suggests that the G10-G45 tertiary base pair is disrupted during the methyl-transfer reaction mediated by Trm11-Trm112.

### 2.7. Tranfer RNAs with Short Variable Region Are Not Methylated by Trm11-Trm112 Complex

**Figure 7:**
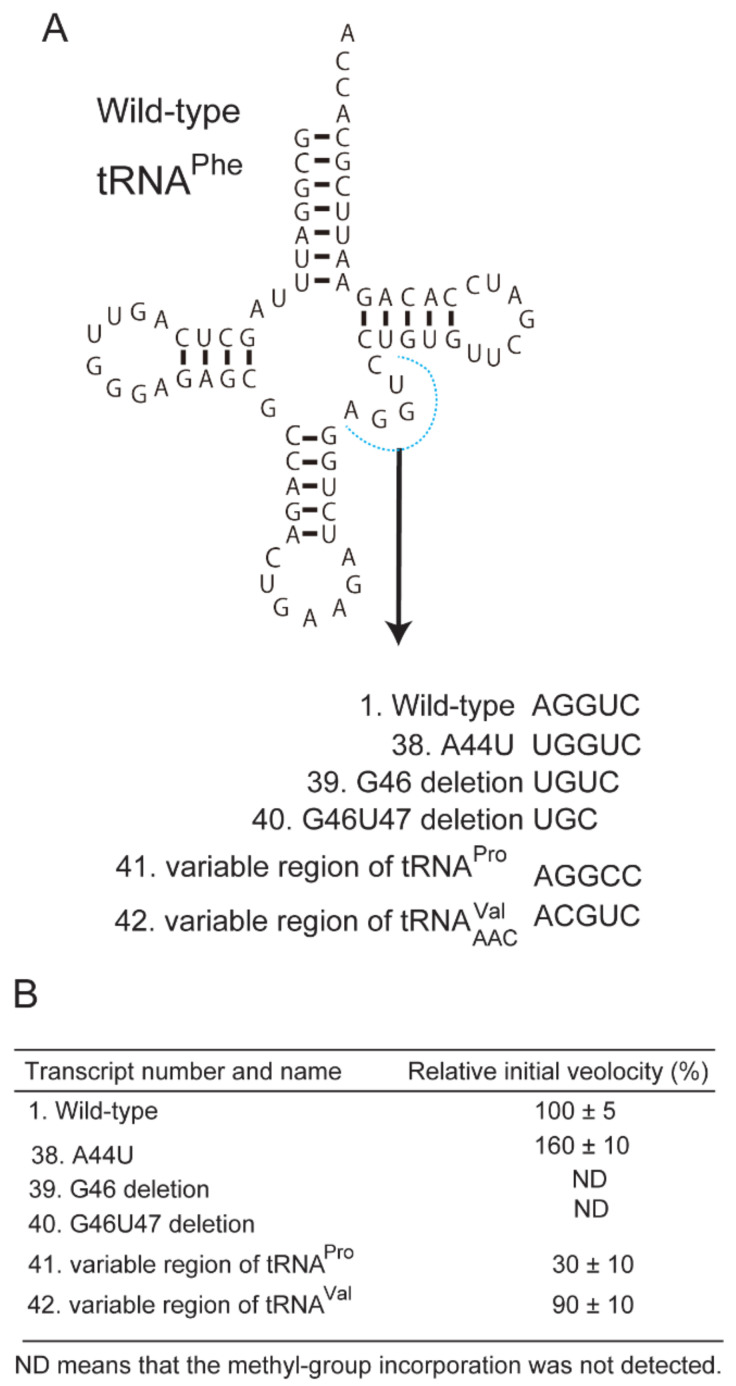
(**A**) tRNA transcripts with a short variable region are not methylated by Trm11-Trm112. Mutations were introduced into the variable region of wild-type tRNA^Phe^ transcript. In the case of transcript 38, A44 in the tRNA^Phe^ transcript was substituted by U. Transcripts 39 and 40 are deletion mutants of the tRNA^Phe^ transcript. In the case of transcripts 41 and 42, the variable region (AGGUC) of tRNA^Phe^ was substituted by the variable region of tRNA^Pro^ (AGGCC) and tRNA^Val^_AAC_ (ACGUC), respectively. (**B**) The initial velocity of methyl-transfer to the wild-type tRNA^Phe^ transcript is expressed as 100%. The data in this figure are averages of the results from four independent experiments. “ND” means that methyl-transfer was not detected.

In the L-shaped tRNA, the variable region makes contact with the D-arm, which contains the methylation site G10 (Figure 6A). To address the effect of the variable region on methylation by Trm11-Trm112, we prepared five tRNA^Phe^ variants (transcripts 38–42 in Figure 7). When A44 was substituted by U, the methyl group acceptance activity was slightly increased. This result shows that the formation of the G26-U44 base pair does not inhibit methylation by Trm11-Trm112. In contrast, when the length of the variable region was shortened, the methyl-group acceptance activity was completely lost (transcripts 39 and 40). This result explains a part of the m^2^G10 modification patterns observed in *S. cerevisiae* tRNAs (Figure 2). Six tRNA species (tRNA^Asp^, tRNA^Glu^, two tRNA^Gly^ and two tRNA^His^) in *S. cerevisiae* tRNAs possess a short variable region, which is composed of three or four nucleotides, and these tRNAs do not possess the m^2^G10 modification. Thus, the presence of a short variable region in tRNA acts as a negative element for methylation by Trm11-Trm112. Furthermore, we investigated whether the sequence of the variable region might have an effect on methylation by Trm11-Trm112. The variable region of tRNA^Phe^ was replaced by that of tRNA^Pro^ or tRNA^Val^_AAC1_ (transcripts 41 and 42 in Figure 7): tRNA^Pro^ and tRNA^Val^_AAC1_ are reported to possess unmodified G10 (Figure 2). Contrary to our expectation, these tRNA transcript variants were methylated by Trm11-Trm112. Thus, Trm11-Trm112 does not distinguish between these sequences of the variable region.

### 2.8. Anticodon-Loop Sequence Is Important for Methylation by Trm11-Trm112

**Figure 8:**
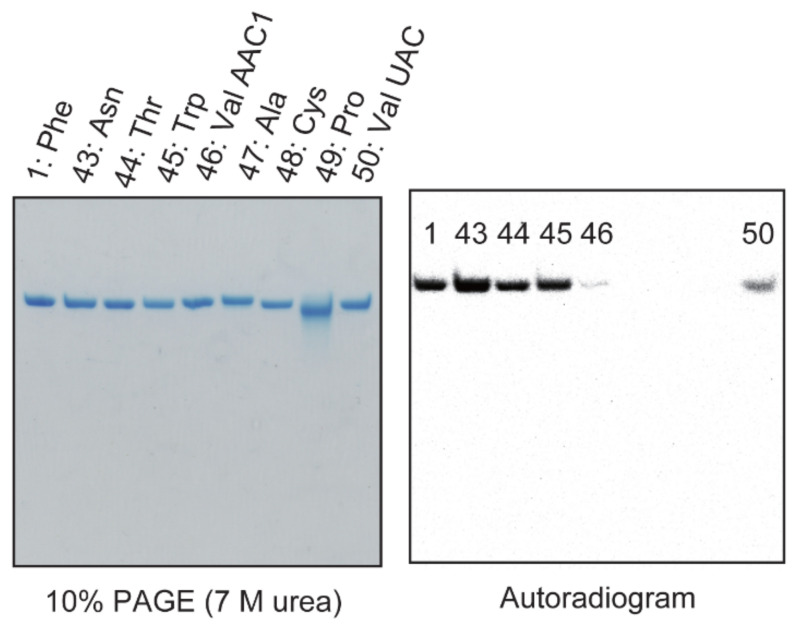
Methylation of nine tRNA transcripts by Trm11-Trm112. Nine tRNA (tRNA^Phe^, tRNA^Asn^, tRNA^Thr^, tRNA^Trp^, tRNA^Val^_AAC1_, tRNA^Ala^, tRNA^Cys^, tRNA^Pro^ and tRNA^Val^_UAC_) transcripts were incubated with Trm11-Trm112 and ^14^C-AdoMet, and 0.025 A260 units of each tRNA were loaded onto a 10% polyacrylamide gel containing 7 M urea. After electrophoresis, an autoradiogram of the gel was obtained. Because the methyl-transfer reaction was stopped after a 2 min, the data in the autoradiogram show the relative initial velocities.

Transfer RNAs, which possess U25 and/or a short variable region, are not methylated by Trm11-Trm112. However, the m^2^G modification patterns of the three tRNA molecular species (tRNA^Ala^, tRNA^Cys^ and tRNA^Val^_AAC1_) shown in Figure 2 are not explainable by these properties because these tRNAs possess C25 and a regular size variable region, which is composed of five nucleotides. To verify whether these tRNA transcripts are substrates for Trm11-Trm112, we prepared the tRNA^Asn^, tRNA^Thr^, tRNA^Trp^, tRNA^Val^_AAC1_, tRNA^Ala^, tRNA^Cys^, tRNA^Pro^ and tRNA^Val^_UAC_ transcripts and tested their methyl-group acceptance activities (Figure 8). The results with the tRNA^Asn^, tRNA^Thr^, tRNA^Trp^, tRNA^Ala^, tRNA^Cys^, tRNA^Pro^ and tRNA^Val^_UAC_ transcripts consisted of their in vivo m^2^G10 modification patterns (Figure 2): the tRNA^Asn^, tRNA^Thr^, tRNA^Trp^ and tRNA^Val^_UAC_ transcripts were methylated by Trm11-Trm112 but tRNA^Ala^, tRNA^Cys^ and tRNA^Pro^ were not methylated. However, unexpectedly, we found that tRNA^Val^_AAC1_ was methylated very slowly. In Figure 8, the slow methylation of tRNA^Val^_AAC1_ is visualized using the gel assay (transcript 46). Therefore, we decided that tRNA^Val^_AAC1_ should be analyzed further, and these experiments are described later in this paper.

### 2.9. U38 in tRNA^Ala^ Is a Negative Element for Methylation by Trm11-Trm112

The presence of an intron disturbs the methylation by Trm11-Trm112 (transcript 2 in Figure 3). This result suggests that Trm11-Trm112 recognizes the anticodon-loop. For tRNA^Ala^ and tRNA^Cys^, we considered whether the sequence of anticodon-loop might act negatively in methylation by Trm11-Trm112.

**Figure 9:**
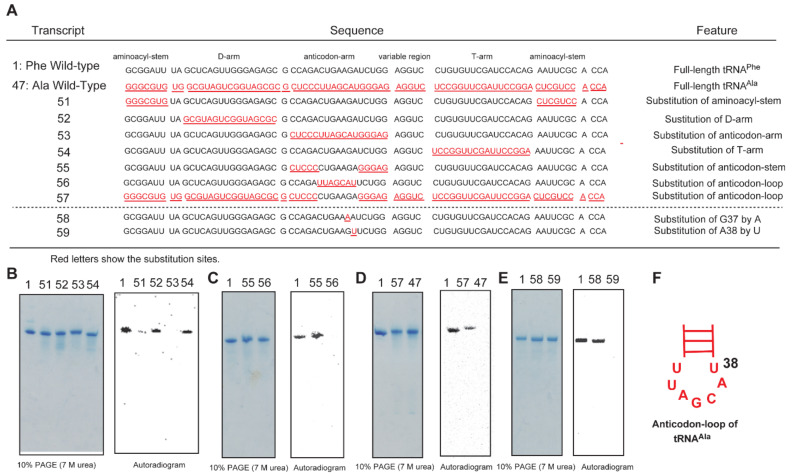
U38 in tRNA^Ala^ acts negatively in methylation by Trm11-Trm112. (**A**) Sequences of chimera tRNA transcripts. The sequence of tRNA^Phe^ (black) was replaced by that of tRNA^Ala^ (red and underlined). (**B**–**E**) The chimera tRNA transcripts were incubated with Trm11-Trm112 and ^14^C-AdoMet and separated by 10% PAGE (7 M urea). Autoradiograms of the gels were obtained. (**F**) The anticodon-loop of tRNA^Ala^. Position 38 is marked.

We prepared nine mutant tRNA transcripts to investigate the anticodon-loop of tRNA^Ala^ (transcripts 51–59 in Figure 9). The sequences of mutant tRNA transcripts are shown in Figure 9A. Transcripts 51–57 are chimera tRNA transcripts of tRNA^Phe^ (transcript 1, black) and tRNA^Ala^ (transcript47, red). As shown in Figure 8, the wild-type tRNA^Ala^ transcript is not methylated by Trm11-Trm112. When the aminoacyl-stem (transcript 51), D-arm (transcript 52) or T-arm (transcript 54) of tRNA^Phe^ was replaced by the corresponding region of tRNA^Ala^, methylation by Trm11-Trm112 could be clearly observed (Figure 9B). In contrast, when the anticodon-arm of tRNA^Phe^ was replaced with that of tRNA^Ala^, the methyl group acceptance activity was completely lost (transcript 53 in Figure 9B). Thus, this result shows that the anticodon-arm of tRNA^Ala^ contains a negative element(s). To clarify whether the anticodon-loop contains the negative element(s), we prepared two chimera tRNA transcripts. When the anticodon-stem of tRNA^Phe^ was replaced with that of tRNA^Ala^, methylation by Trm11-Trm112 was still observed (transcript 55 in Figure 9C). In contrast, the substitution of the anticodon-loop of tRNA^Phe^ with that of tRNA^Ala^ caused complete loss of methyl group acceptance activity (transcript 56 in Figure 9C). Furthermore, when the anticodon-loop of tRNA^Ala^ was substituted with that of tRNA^Phe^ (transcript 57), this transcript was clearly methylated (Figure 9D). Thus, these results demonstrate that the negative element(s) for methylation by Trm11-Trm112 in tRNA^Ala^ is contained in the anticodon-loop. In general, the base at position 38 in tRNA is conserved as purine. However, in the case of tRNA^Ala^, position 38 is U. We prepared two point-mutation tRNA^Phe^ transcripts (transcripts 58 and 59). When G37 in tRNA^Phe^ was substituted by A, this transcript was well methylated (transcript 58 in Figure 9E). In contrast, when A38 in tRNA^Phe^ was substituted by U, this transcript was not methylated (transcript 59 in Figure 9E). Taking these results together, we conclude that U38 in tRNA^Ala^ (Figure 9F) is a negative element for methylation by Trm11-Trm112.

### 2.10. The U32-A38 Base Pair in tRNA^Cys^ Is a Negative Element for Methylation by Trm11-Trm112

**Figure 10:**
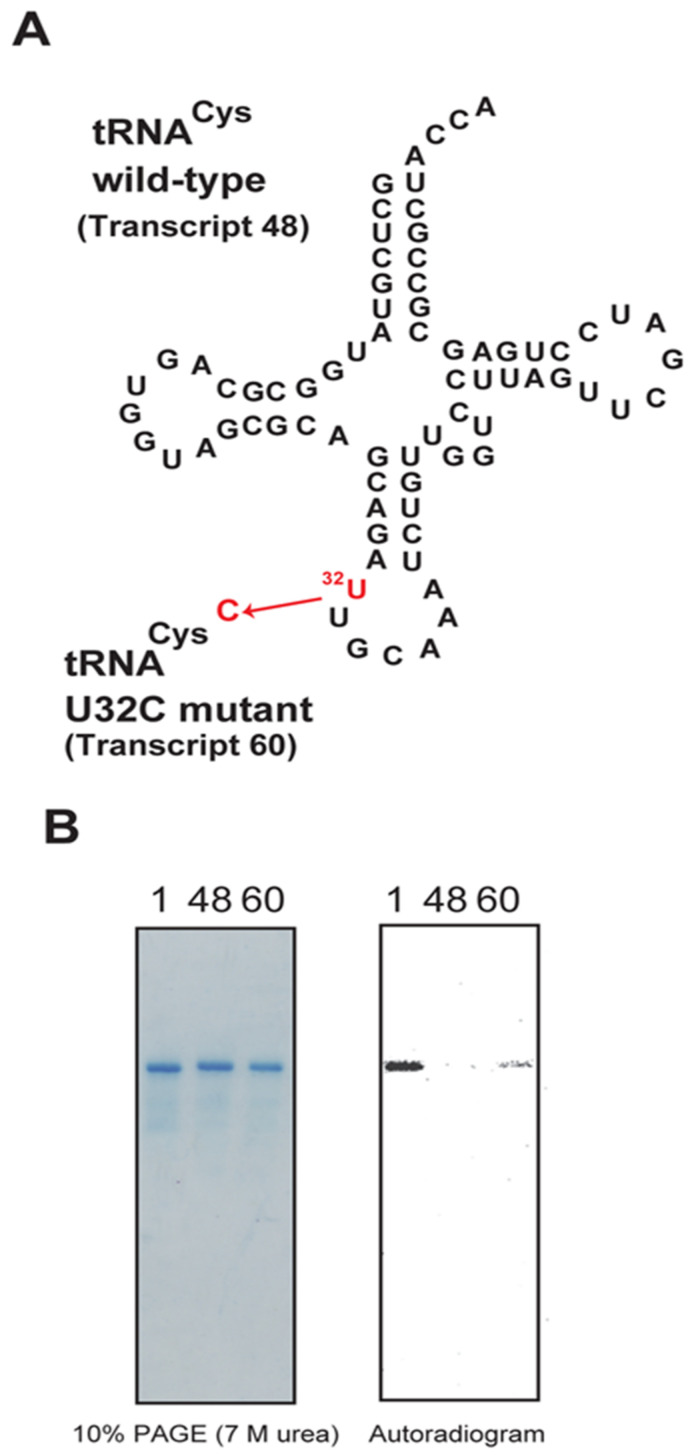
Acceptances of the methyl groups of the wild-type and mutant tRNA^Cys^ transcripts by the Trm11-Trm112 complex were compared. (**A**) The sequence of tRNA^Cys^ is represented in the cloverleaf structure. The U32 was replaced by C (transcript 60). (**B**) Acceptances of the methyl group of tRNA^Phe^, tRNA^Cys^ and mutant tRNA^Cys^ transcripts are compared.

Trm11-Trm112 recognizes the anticodon-loop in tRNA. The anticodon-loop of tRNA^Cys^ is not standard because U32-A38 and U33-A37 base pairs can be formed (Figure 10A). We considered that these unusual base pairs may act negatively for methylation by Trm11-Trm112. To test this idea, we prepared a point mutation tRNA^Cys^ transcript, in which U32 was substituted by C (transcript 60). Consistent with our hypothesis, as shown in Figure 10B, transcript 60 was methylated by Trm11-Trm112. In contrast, the wild-type tRNA^Cys^ transcript (transcript 48) is not methylated. These results show that the U32-A38 base pair is a negative element for methylation by Trm11-Trm112. It should be mentioned that U32 and A37 are modified to Ψ32 and i^6^A37, respectively, in native tRNA. Therefore, the Ψ32-A38 base pair is probably formed in native tRNA^Cys^. The precise effect of the Ψ32-A38 base pair on methylation by Trm11-Trm112 is not clear.

### 2.11. A Portion of tRNA^Val^_AAC1_ Is Methylated by Trm11-Trm112 in S. cerevisiae Cells

**Figure 11:**
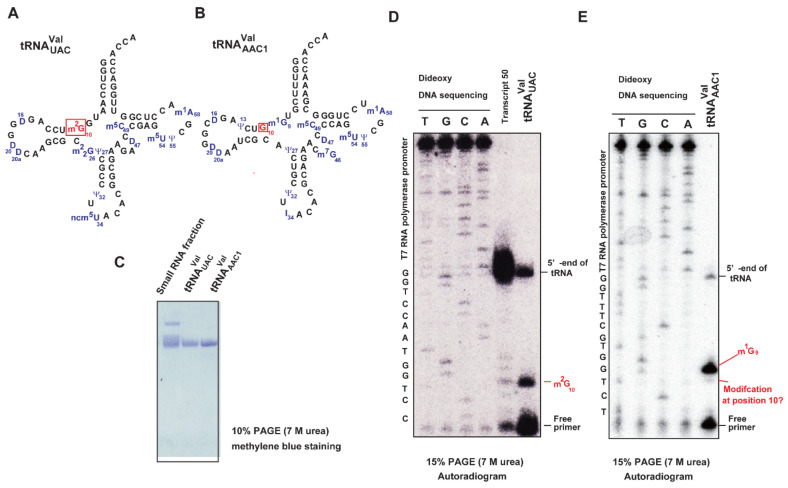
Primer extension experiments suggest that a portion of G10 in tRNA^Val^_AAC1_ is modified. (**A**) Cloverleaf structure of tRNA^Val^_UAC_. m^2^G10 and modified nucleosides are colored in red and blue, respectively. (**B**) Cloverleaf structure of tRNA^Val^_AAC1_. G10 and modified nucleosides are colored in red and blue, respectively. (**C**) Purified tRNA^Val^_UAC_ and tRNA^Val^_AAC1_ (0.05 A260 units each) were analyzed by 10% PAGE (7 M urea). The gel was stained with methylene blue. (**D**) Purified tRNA^Val^_UAC_ was analyzed using the primer extension method. Because dideoxy DNA sequencing was preformed using the template DNA for transcript 50, the T7 RNA polymerase promoter region can be read in addition to the tRNA coding region. Transcript 50 was methylated by Trm11-Trm112 with non-radioisotope AdoMet and then used for the primer extension. The paused band derived from m^2^G10 is marked. (**E**) Purified tRNA^Val^_AAC1_ was analyzed using the primer extension method. In addition to the paused band derived from m^1^G9, a faint posed band is observed at position 10.

**Figure 12:**
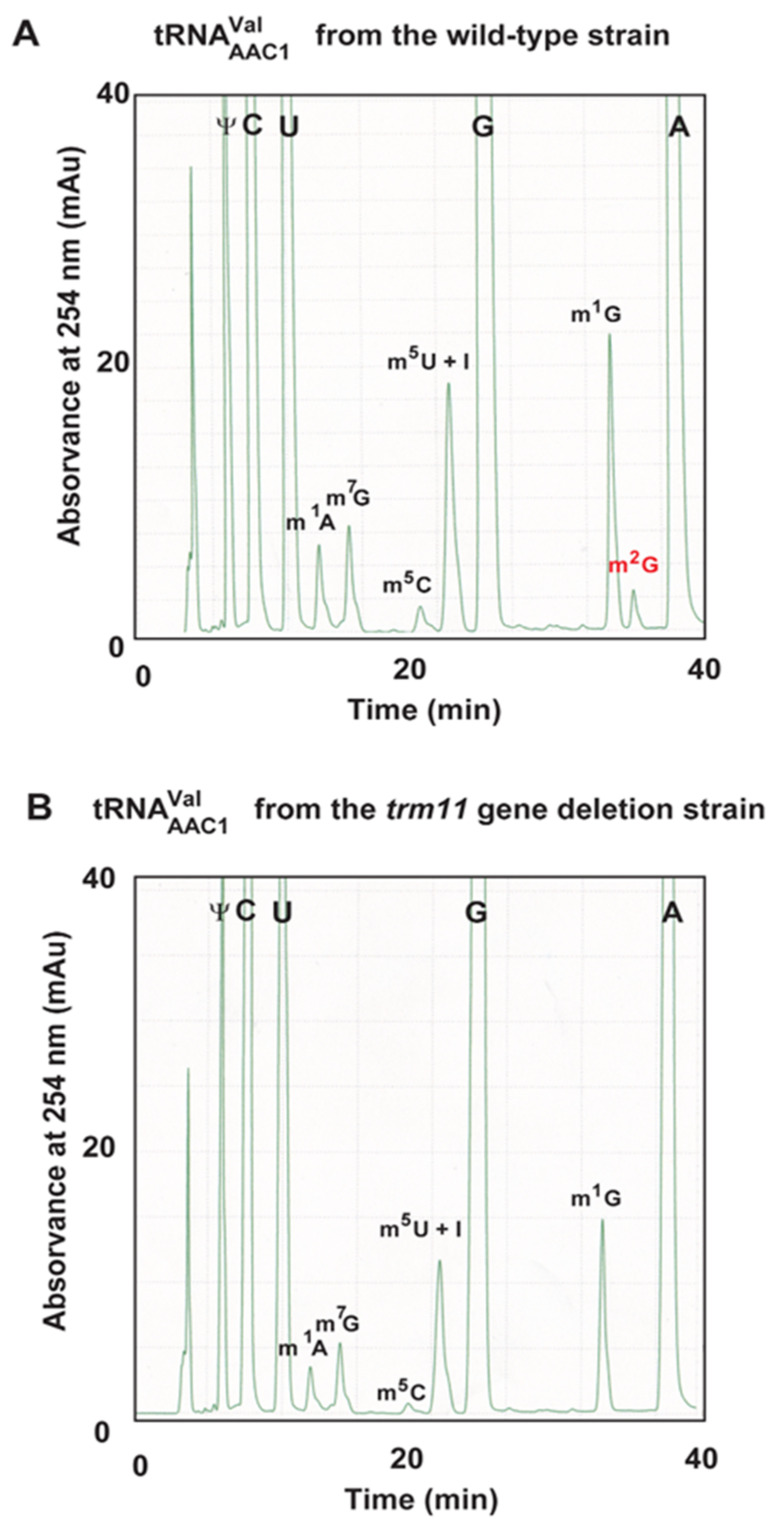
The m^2^G nucleoside is present in tRNA^Val^_AAC1_ from the wild-type *S. cerevisiae* strain but not in tRNA^Val^_ACC1_ from the *trm11* gene deletion strain. (**A**) Purified tRNA^Val^_AAC1_ (0.60 A260 units) from the wild-type *S. cerevisiae* strain was digested to nucleosides, and then, the modified nucleosides were analyzed. The peak of m^2^G is highlighted in red. The peaks of m^5^U and I overlap. D is not visible because D does not absorb ultra-violet light at 254 nm. (**B**) Modified nucleosides in purified tRNA^Val^_AAC1_ (0.45 A260 units) from the *trm11* gene deletion strain were analyzed. The peak corresponding to m^2^G is not observed.

Our biochemical studies on Trm11-Trm112, as described in this report, explain almost all of the m^2^G10 modification patterns observed in *S. cerevisiae* class I tRNAs: class I tRNAs possess regular-size (5 nt) or shorter variable regions. However, one exception remained: tRNA^Val^_AAC1_. Although tRNA^Val^_AAC1_ is reported to possess unmodified G10 [50], our experiments show that this tRNA transcript (transcript 46) is slowly methylated by Trm11-Trm112 (Figure 8). We hypothesized that this tRNA may be methylated by Trm11-Trm112 in *S. cerevisiae* cells. To confirm this idea, we purified native tRNA^Val^_UAC_ (Figure 11A) and tRNA^Val^_AAC1_ (Figure 11B) using the solid-phase DNA probe method [51,52]. We prepared a small RNA fraction using Q-Sepharose column chromatography from which native tRNA^Val^_UAC_ and tRNA^Val^_AAC1_ were purified (Figure 11C). Initially, we performed primer extension experiments to verify whether G10 is modified. The primer was designed to be complimentary from position36 to position 14 in tRNA. It should be mentioned that m^2^G10 is detectable using the primer extension experiment, although m^2^G can form a Watson–Crick base pair with C. To show this, we used native tRNA^Val^_UAC_ as a positive control: this tRNA possesses m^2^G10 (Figure 11A). As shown in Figure 11D, m^2^G10 in tRNA^Val^_UAC_ paused the reverse transcriptase reaction. Next, we analyzed tRNA^Val^_AAC1_ in the same experimental condition. In addition to the paused band derived from m^1^G9, a faint band was observed at position 10 (Figure 11E). This result suggests that a portion of G10 in tRNA^Val^_AAC1_ is modified. In *S. cerevisiae* tRNAs, m^2^G is only one modification at position 10 (Figure 2). Therefore, we considered that a portion of tRNA^Val^_AAC1_ is methylated by Trm11-Trm112 in vivo. To confirm this idea, we purified tRNA^Val^_AAC1_ from wild-type and *trm11* gene deletion strains of *S. cerevisiae*. The modified nucleosides in these tRNAs were analyzed (Figure 12). In tRNA^Val^_AAC1_ from the wild-type strain, a small but clear m^2^G peak was observed (Figure 12A). In contrast, tRNA^Val^_AAC1_ from the *trm11* gene deletion strain does not contain m^2^G (Figure 12B). Thus, the m^2^G modification in tRNA^Val^_AAC1_ from the wild-type strain is derived from the activity of Trm11-Trm112. Taking these experimental results together, we conclude that a portion of tRNA^Val^_ACC1_ is modified by Trm11-Trm112 in vivo. The modification pattern of tRNA^Val^_AAC1_ in *S. crevisiae* cells coincides with the in vitro experimental result (Figure 8). The ID number of tRNA^Val^_AAC1_ from *S. crevisiae* in the T-psi-C database [43] is tdbR00000464. Our experimental results in this study provide novel additional information about this tRNA. When the peak area of m^1^G of tRNA^Val^_AAC1_ from the wild-type strain was expressed as 100%, the peak area of m^2^G was calculated as 13% (Figure 12A). Therefore, more than 85% of tRNA^Val^_AAC1_ possesses unmodified G10, as reported previously [50].

## 3. Discussion

For a long time, it has been an enigma why yeast Trm11-Trm112 acts only on a subset of tRNAs. In this study, our biochemical experiments explain the m^2^G10 modification pattern of all class I tRNAs from *S. cerevisiae*. However, two class II tRNAs (tRNA^Leu^ and tRNA^Ser^) were not analyzed. tRNA^Leu^ has the m^2^G10 modification but tRNA^Ser^ does not (Figure 2). Transfer RNA^Ser^ species possess a unique Um44 modification, which is formed by Trm44 [53]. Therefore, there is a possibility that the Um44 modification has a negative effect on m^2^G10 formation by Trm11-Trm112. In this study, we used tRNA transcripts that do not possess modifications. Therefore, we did not investigate class II tRNAs. With tRNA modifications, one modification often affects the formation of other modifications either negatively or positively [2,54]. For example, in the case of *S. cerevisiae*, the D20 modification mediated by Dus2 [55,56] was not formed in the absence of the Gm18 modification [57]. Furthermore, recently, the order of the modifications (m^5^U54, Ψ55 and m^1^A58) of the T-arm of tRNA^Phe^ has been reported [58]: in yeast cytoplasmic tRNAs, m^5^U54, Ψ55 and m^1^A58 are formed by Trm2 [59,60], Pus4 [61] and the Trm6-Trm61 complex [62], respectively. Moreover, with the anticodon-loop modifications, the modified nucleosides and tRNA modification enzymes form circuits (networks) [63,64,65,66]. For example, the Gm34 modification in tRNA^Phe^ mediated by the Trm7-Trm734 complex [63,66,67,68] requires Cm32 modification by the Trm7-Trm732 complex [68] and the m^1^G37 modification by Trm5 [69,70,71]. In this view point, the Ψ13 modification, which is synthesized by Pus7 [72,73], may have an effect on the methylation by Trm11-Trm112 because various tRNAs containing Ψ13 do not possess the m^2^G10 modification. In addition, the anticodon-loop region contains various hyper modifications (Figure 2 and Reference [1]). Because Trm11-Trm112 recognizes the nucleotide around position 38, these modifications in the anticodon-loop may affect methylation by Trm11-Trm112. To clarify these issues, further study will be necessary.

During the course of this study, Bourgeois et al. proposed a model of interaction between Trm11-Trm112 and tRNA [9]. In their model, Trm112 interacts with the anticodon-loop region in a substrate tRNA according to the movement of the THUMP domain in Trm11. Our biochemical experimental results described in the current study support this model because Trm11-Trm112 recognizes the nucleotide around position 38. To clarify the molecular details, a crystal structure of a Trm11-Trm112 and tRNA complex is required.

The physiological role of the m^2^G10 modification of tRNA in eukaryotic cells is not clear although m^2^G10 probably stabilizes the D-stem structure. In fact, human disease derived from a defect of m^2^G10 modification has not been reported [74]. A double deletion strain of the *S. cerevisiae trm1* and *trm11* genes showed growth retardation [4]. Therefore, the m^2^G10 modification probably works coordinately with other modifications such as m^2^_2_G26. A similar phenomenon has been reported: a double mutant strain of the *S. cerevisiae trm4* and *trm8* genes showed temperature-sensitive growth [75]. Trm4 and Trm8 is a multisite-specific tRNA m^5^C methyltransferase [76] and the catalytic subunit of tRNA m^7^G46 methyltransferase [77], respectively. Thus, in *S. cerevisiae* cells, the modifications in the three-dimensional core of tRNA probably works coordinately. Trm112 is a hub protein that activates multiple methyltransferases [6,26]. Therefore, the formation of the Trm11 and Trm112 complex has effects on the activities of other methyltransferases in *S. cerevisiae* cells. If the amount of Trm11 is increased, the activities of other methyltransferases are downregulated, and this effect is not negligible. To understand the physiological role of the m^2^G10 modification and Trm11-Trm112, further study is necessary.

## 4. Conclusions

**Figure 13:**
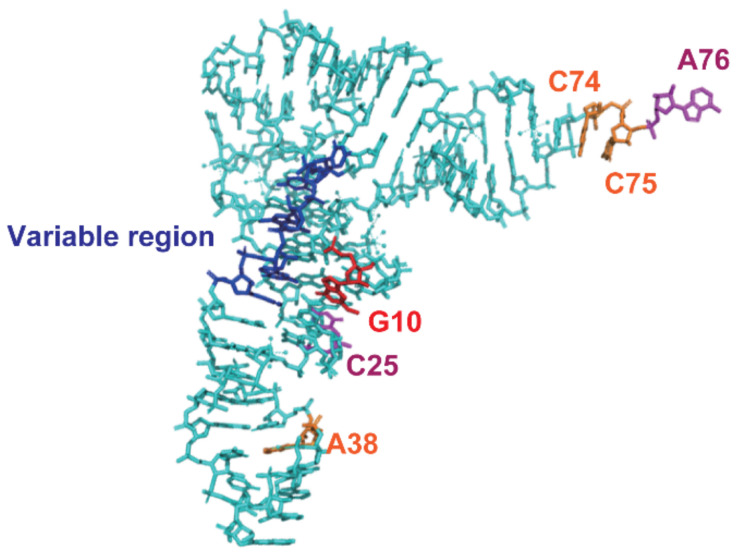
The elements required for methylation by Trm11-Trm112 are marked on the stick model of tRNA^Phe^ structure. The methylation site (G10) is highlighted in red. The essential elements, A76 and C25, are colored in magenta. Trm11-Trm112 methylates tRNAs, which possess a regular size variable region (blue). U38 in tRNA^Ala^ and U32-A38 base pair in tRNA^Cys^ act negatively on the methylation by Trm11-Trm112. To show the position 38, A38 in tRNA^Phe^ is colored in orange.

In this study, we clarified the elements in class I tRNAs from *S. cerevisiae* required for methylation by Trm11-Trm112. The results are summarized in Figure 13. Precursor tRNA does not act as a substrate for Trm11-Trm112. The presence of a 5′-leader sequence, intron and 3′-trailer sequence negatively affect methylation by Trm11-Trm112. The CCA terminal region is essential for the methylation by Trm11-Trm112. In the CCA terminus, A76 (magenta in Figure 13) is essential but C74 and C75 (orange in Figure 13) are not required for methylation by Trm11-Trm112. Furthermore, a deletion of the CCA terminus causes loss of methylation by Trm11-Trm112. Therefore, the phosphate ribose backbone of C74 and C75 region is required for methylation by Trm11-Trm112. Trm11-Trm112 methylates standard tRNAs. Transfer RNAs that possess a regular-size variable region (5 nt: blue in Figure 13) are methylated by Trm11-Trm112. Therefore, six tRNA species (tRNA^Asp^, tRNA^Glu^, two tRNA^Gly^, and two tRNA^His^), which possess short variable regions, are not substrates for Trm11-Trm112. Trm11-Trm112 requires the G10-C25 base pair for methylation. In Figure 13, G10 and C25 are colored in red and magenta, respectively. Therefore, five tRNAs (tRNA^Glu^, two tRNA^Gly^ and two tRNA^Pro^) that possess the G10-U25 base pair are not methylated by Trm11-Trm112. Furthermore, Trm11-Trm112 recognizes the anticodon-loop region. Transfer RNA^Ala^ and tRNA^Cys^ possess a non-standard anticodon-loop. In the case of tRNA^Ala^, U38 acts negatively in methylation by Trm11-Trm112. In the case of tRNA^Cys^, the unusual U32-A38 base pair disturbs the methylation by Trm11-Trm112. In *S. cerevisiae*, tRNA introns are inserted between positions 38 and 39. Therefore, these experimental results suggest that Trm11-Trm112 interacts with the nucleotide around position 38. The interaction between the T-arm and D-arm is probably required for the maintenance of the correct distances and angles between the CCA terminus, anticodon-loop and methylation site (G10). As shown in Figure 13, Trm11-Trm112 probably interacts with substrate tRNA from the inside of the L-shaped tRNA structure. In addition, our in vitro experiment showed that the tRNA^Val^_AAC1_ transcript was slowly methylated by Trm11-Trm112. However, position 10 in this tRNA was reported to be unmodified G. We purified tRNA^Val^_AAC1_ from the wild-type and *trm11* gene deletion strains and confirmed that a portion (10–13%) of tRNA^Val^_AAC1_ is methylated by Trm11-Trm112 in *S. cerevisiae* cells. Thus, our biochemical studies described in this report explain the m^2^G10 modification pattern of all class I tRNAs from *S. cerevisiae* and elucidates the Trm11-Trm112 binding sites in tRNA.

## 5. Materials and Methods

### 5.1. Materials

[Methyl-^14^C]-AdoMet (1.95 GBq/mmol) and [methyl-^3^H]-AdoMet (2.89 TBq/mmol) were purchased from ICN. Non-radioisotope-labeled AdoMet was obtained from Sigma, Tokyo, Japan. Ni-NTA super-flow was purchased from Qiagen (Tokyo, Japan). Superdex 75 Preparation Grade and Q-Sepharose Fast Flow were bought from GE Healthcare. DNA oligomers were bought from Invitrogen. [γ-^32^P]-ATP (222 TBq/mmol) was purchased from Perkin-Elmer Japan, Tokyo, Japan. All other chemical reagents were of analytical grade.

### 5.2. Construction of Trm11-Trm112 Expression System in E. coli Cells

Cloning of *trm11* and *trm112* genes has been previously reported [45]. Briefly, the *trm112* gene was fused to the His x 6 tag and HRV3C protease cleavage site in pET21a (Novagen, Birmingham, UK). The *trm11* gene with T7 RNA polymerase promoter was inserted down-stream from the *trm112* gene. The constructed plasmid was named pET21a-6x His-Trm112-Trm11.

### 5.3. Expression of Trm11-Trm112 in E. coli Cells

The pET21a-6x His-Trm112-Trm11 plasmid was introduced into *E. coli* BL21(DE3) Rosetta 2 strain. The transformant was cultured at 37 °C in 1 L of LB medium containing 100 µg/mL ampicillin until the optical density at 600 nm reached 0.6. The culture was cooled on ice for 1 h, and then, isoproypl-β-D-thiogalactopyranoside and ZnCl_2_ were added (final concentrations of 1 mM and 100 µM, respectively). The culture was further incubated at 20 °C for 48 h. The cells were collected by centrifugation at 4320× *g* at 4 °C for 20 min, frozen in liquid nitrogen and stored at −80 °C before use.

### 5.4. Purification of Trm11-Trm112

Wet cells (3.0 g) were suspended in 15 mL buffer A (50 mM Tris-HCl (pH 7.6), 5 mM MgCl_2_, 200 mM KCl, 20 mM imidazole) supplemented with Halt protease inhibitor single-use cocktail (Thermo Fisher Scientific, Tokyo, Japan) and disrupted with an ultrasonic disruptor (model VCX-500, Sonics and Materials. Inc, CT, USA). The supernatant was collected by centrifugation at 38,900× *g* at 4 °C for 20 min and then loaded onto a Ni-NTA super-flow column (5 mL) equilibrated with buffer A. After the unbound proteins were washed off with buffer A, the bound proteins were eluted stepwise using buffer A containing 500 mM imidazole. Trm11-Trm112 elution fractions were assessed by 15% SDS-PAGE and combined. Then, 100 µL of HRV3C protease (Takara, Kyoto, Japan, code: 7360) was added to the sample (10 mL). The sample was dialyzed against buffer B (50 mM Tris-HCl (pH 7.6), 5 mM MgCl_2_, 10 µM ZnCl_2_, 1 mM 2-mercaptoethanol, 50 mM KCl, 5% glycerol) at 4 °C for 12 h. The dialyzed sample was loaded onto a Ni-NTA super-flow column again to remove any undigested samples and HRV3C protease. The flow-through and wash fractions were combined, and the sample volume was reduced to 5 mL in a Vivaspin 15R filter device (Millipore, Tokyo, Japan; Mw CO, 10,000). The sample was loaded onto a Superdex 75 Preparation Grade column (120 mL) equilibrated with buffer C (50 mM Tris-HCl (pH 7.6), 5 mM MgCl_2_, 10 µM ZnCl_2_, 6 mM 2-mercaptoethanol, 200 mM KCl, 5% glycerol). The eluted Trm11-Trm112 fractions were assessed using 15% SDS-PAGE, combined and concentrated in a Vivaspin 15R filter device. The concentrated sample was added glycerol (final concentration 50%) and stored at −30 °C.

### 5.5. Preparation of tRNA Transcripts

Transfer RNA transcripts were prepared using an in vitro T7 RNA polymerase reaction, as described previously [78]. We used 2′-*O*-methylated DNA primers for construction of template DNAs. For example, the template DNA for tRNA^Phe^ transcript was constructed using the following primers: tRNA^Phe^ forward primer, 5′-GGG TAA TAC GAC TCA CTA TAG CGG ATT TAG CTC AGT TGG GAG CGC CAG ACT GAA GAT CTG GAG GTC -3′; tRNA^Phe^ reverse primer, 5′-TmGG TGC GAA TTC TGT GGA TCG AAC ACA GGA CCT CCA GAT CTT CAG TCT GG-3′. The transcripts were purified using Q-Sepharose column chromatography and 10% PAGE (7 M urea).

### 5.6. Measurement of Activity of Trm11-Trm112

Typical assay conditions were as follows: 0.10 µM Trm11-Trm112, 10.0 µM tRNA transcript and 20.1 µM AdoMet (20 mM non-radioisotope labeled AdoMet and 0.06 mM ^3^H-AdoMet) in 30 µL of buffer C were incubated at 30 °C for 2 min, and then, 20 µL of sample was used for the filter assay. To visualize the methylation, ^14^C-AdoMet was used. Then, 0.10 µM Trm11-Trm112, 10.0 µM tRNA transcript and 20.0 µM ^14^C-AdoMet in 30 µL of buffer C were incubated at 30 °C for 2 min, and then, RNA was recovered by phenol-chloroform treatment and ethanol precipitation. The RNA was dissolved in 20 µL of water, and then, 0.025 A260 units of RNA was loaded onto a 10% polyacrylamide gel containing 7 M urea. After the electrophoresis, the gel was stained with methylene blue and dried. The incorporation of the ^14^C-methyl groups was monitored with a FLA-2000 (GE Healthcare, Tokyo, Japan) imaging analyzer.

### 5.7. Sacharomyces cerevisiae Strains and Culture

A block of wild-type *S. cerevisiae* (a block of baker’s yeast, 200 g) was purchased from a local bakery in Japan. The *Saccharomyces cerevisiae trm11* gene deletion strain was purchased from Funakoshi (Tokyo, Japan, code: YSC6273-201937501). The *trm11* gene deletion strain was cultured in 1 L of YPD medium at 30 °C for 10 h, and then, the cells were collected by centrifugation at 4320× *g* at 4 °C for 30 min. The cells were frozen by liquid nitrogen and stored at −80 °C before use.

### 5.8. Preparation of Small RNA Fraction from S. cerevisiae Cells

Wet cells (50 g) were suspended in 250 mL TE buffer (10 mM Tris-HCl (pH 8.0) and 1 mM EDTA), and then, 250 mL TE buffer-saturated phenol was added. The suspension was shaken at 37 °C for 2 h and then centrifuged at 6000× *g* at 4 °C for 10 min. The aqueous-phase was collected, and then, an equal volume (200 mL) of TE buffer-saturated phenol was added. The sample was shaken at 37 °C for 30 min and then centrifuged at 6000× *g* at 4 °C for 10 min. Total RNA was prepared from the aqueous-phase (200 mL) by ethanol precipitation. The total RNA was dissolved in 40 mL of buffer D (20 mM Tris-HCl (pH 7.6), 400 mM NaCl) and loaded onto a 10 mL Q-Sepharose column. RNAs were separated by a 400–1000 mM NaCl linear-gradient in buffer D. The small RNA fractions were assessed by 10% PAGE (7 M urea), combined and recovered by ethanol precipitation.

### 5.9. Purification of tRNA^Val^_UAC_ and tRNA^Val^_AAC1_ Using the Solid-Phase DNA Probe Method

Native tRNA^Val^_UAC_ and tRNA^Val^_AAC1_ were purified from the small RNA fraction using the solid-phase DNA probe method, as reported previously [52]. Hybridization of tRNA and DNA probe was performed by cooling from 69 °C to 65 °C over 10 min. The sequences of DNA probes are as follows: for tRNA^Val^_UAC_, 5′-GTA AAG GCG ATG TCT TGA ACC AC-biotin 3′; for tRNA^Val^_AAC1_, 5′-GTTAAG CAG ATG CCA TAA CCG AC-biotin 3′. The probes were designed to be complementary from position 36 to position 13 in the tRNA. Because tRNA^Val^_UAC_ possesses m^2^_2_G26, we used T instead of C as the corresponding complementary sequence.

### 5.10. Primer Extension

Primer sequences for primer extension are as follows: for tRNA^Val^_UAC_, 5′-GTA AAG GCG ATG TCT TGA ACC ACT-3′; for tRNA^Val^_AAC1_, 5′-GTC AAG CAG ATG CCA TAA CCG ACT-3′. The primers were purified by 10% PAGE (7 M urea) before 5′-^32^P-labeling to remove short byproducts. Then, 50 pmol of the primer was incubated with 20 units of T4 polynucleotide kinase (Takara, Kyoto, Japan) and 1 µL of γ-^32^P-ATP at 37 °C for 1 h. The 5′-^32^P-labeled primer was purified by 10% PAGE (7 M urea) again. Dideoxy DNA sequencing was performed using a Promega dideoxy DNA sequencing kit (Promega, Madison, WI, USA). The template DNAs for T7 RNA polymerase transcription were used as the template DNA for dideoxy DNA sequencing. Then, 0.02 A260 units of purified tRNA^Val^_UAC_ (or tRNA^Val^_AAC1_), 1.0 pmol 5′-^32^P-labeled primer, 1 mM EDTA (pH 8.0) and 1 mM of each dNTP mixture (total volume was 30 µL) were incubated at 90 °C for 5 min to denature the tRNA structure and then cooled on ice. A total of 4 µL of 5 × Prime Script buffer (Takara, Kyoto, Japan), 200 units of Prime Script reverse transcriptase (Takara, Kyoto, Japan, code: 2680A) and 1 µL of RNasin (Promega, RNase inhibitor, code: N2111) were added to the sample. The sample was incubated at 45 °C for 60 min and then heated at 70 °C for 15 min. The reverse transcriptase reaction was stopped by adding 20 µL of the gel loading solution (0.02% xylene cyanole, 0.02% bromophenol blue and 7 M urea). The sample was separated by 15% PAGE (7 M urea). The gel was dried, and the autoradiogram of the gel was obtained using a FLA-2000 (GE Healthcare) imaging analyzer.

### 5.11. Nucleoside Analysis of Purified tRNA

Nucleoside analysis of purified tRNA was performed as described previously [79]. Purified tRNA^Val^_UAC_ (0.60 A260 units) or tRNA^Val^_AAC1_ (0.45 A260 units) were digested with 2 µg RNase A, 4 µg snake venom phosphodiesterase and 0.5 units of bacterial alkaline phosphatase in 20 µL of 50 mM Tris-HCl (pH 8.0) at 37°C for 24 h, and then, nucleosides were analyzed using a Hitachi L-2000 HPLC system equipped with a reverse-phase C18 column (Nucleosil 100 C18: 25 cm × 4.6 mm, 7 µm; GL Science, Inc., Tokyo, Japan). The elution positions of modified nucleosides (m^1^A, m^7^G, m^5^C, I and m^5^U) were confirmed using standard markers (Sigma). The elution times of m^1^G and m^2^G were determined based on enzymatic formation using *Aquifex aeolicus* TrmD [80] and Trm1 [81], respectively.

## Supplementary Materials

The following supporting information can be downloaded at: https://www.mdpi.com/article/10.3390/ijms23074046/s1, supplementary file including Figures S1 and S2.
