# Supplementary material for: Required Elements in tRNA for Methylation by the Eukaryotic tRNA (Guanine-N2-) Methyltransferase (Trm11-Trm112 Complex)
Source: Int J Mol Sci. 2022 Apr 6;23(7):4046. doi: 10.3390/ijms23074046 (PMC8999500; doi:10.3390/ijms23074046)
Supplement: Supplementary file 1 [file ijms-23-04046-s001.zip › ijms-1646530 - supplementary.pdf]

Supplementary file

Required elements in tRNA for methylation by the eukaryotic tRNA (guanine-N^2-) methyltransferase (Trm11-Trm112 complex)

Yu Nishida, Shiho Ohmori, Risa Kakizono, Kunpei Kawai, Miyu Namba, Kazuki Okada, Ryota Yamagami, Akira Hirata † and Hiroyuki Hori*

Department of Materials Science and Biotechnology, Graduate School of Science and Engineering, Ehime University, 3 Bunkyo-cho, Matsuyama 790-8577, Japan

E-mails: yu_nishida_ehime_u@yahoo.co.jp (Y.N.); sh10_2790@yahoo.co.jp (S.O.); risa.kakizono@gmail.com (R.K.); kumpei0405@gmail.com (K.K.); miyu.n.014744687@gmail.com (M.N.); aporon8okada2006@yahoo.co.jp (O.K.); yamagami.ryota.bn@ehime-u.ac.jp (R.Y.); ahirata@tokushima-u.ac.jp (A.H.); hori.hiroyuki.my@ehime-u.ac.jp (H.H.)

† Present address; Department of Natural Science, Graduate School of Technology, Industrial and Social Science, Tokushima University, 2-1 Minamijosanjimacho, Tokushima 770-8506, Japan

* Author to whom correspondence should be addressed; E-Mail: hori.hiroyuki.my@ehime-u.ac.jp
Tel.: +81-89-927-8548

Co-expression of Trm11 and Trm112 in *Escherichia coli* cells and purification

The *trm112* with a His x 6 tag coding region fused at the end of gene was connected upstream of the *trm11* gene and these genes were inserted into the pET21a plasmid vector. The constructed vector, pET21a-*trm112*His x 6 tag-*trm11*, was introduced into *E. coli* BL21 (DE3) Rosetta 2 strain and recombinant proteins were expressed. The complex of Trm11-Trm112-His x 6 tag was purified by NiNTA super-flow column chromatography (Supplementary Figure 1A left lane), and then the His x 6 tag was cleaved by HRV3C protease. The cleaved His x 6 tag and HRV3C protease were removed from the sample by passing through the NiNTA super-flow column again (Supplementary Figure 1A right lane). The sample was further purified by Superdex-75 High Resolution gel-filtration column chromatography as shown in Supplementary Figure 1B.

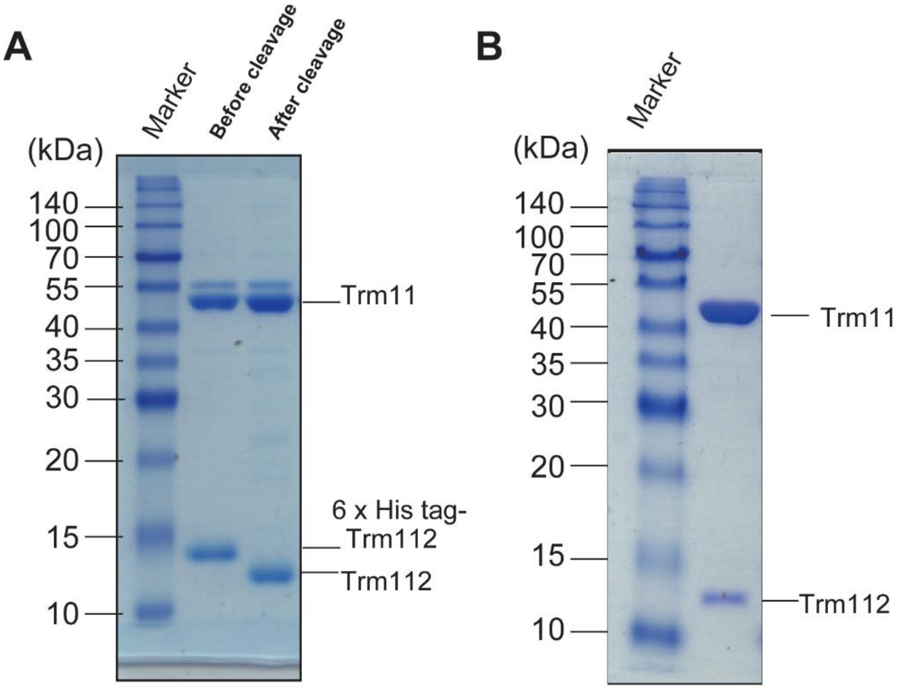

Supplementary Figure S1. Purification of Trm11-Trm112 complex. A After NiNTA super-flow column chromatography (left lane), the His x 6 tag was cleaved by HRV3C protease (right lane). 6.8 μg of each of sample was analyzed by 15% SDS-polyacrylamide gel electrophoresis (PAGE). The gel was stained with Coomassie Brilliant Blue. **B** Purified Trm11-Trm112 (7.4 μg) was analyzed by 15% SDS-PAGE. The gel was stained with Coomassie Brilliant Blue.

Measurement of methyltransferase activity of Trm11- Trm112 complex

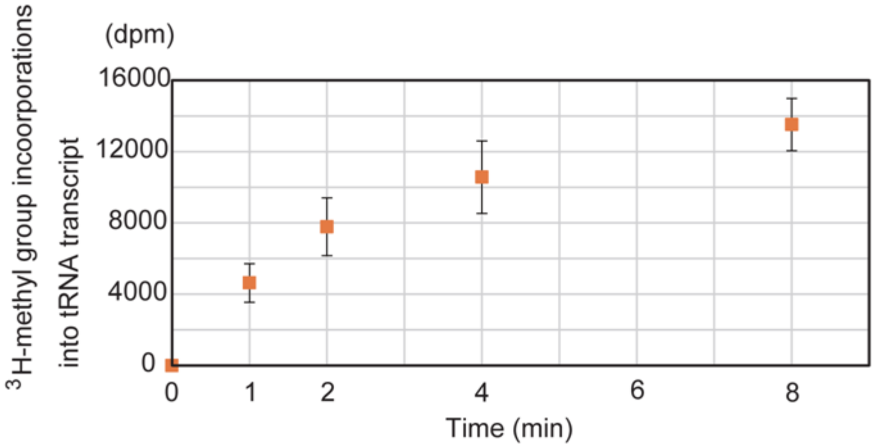

Supplementary Figure S2. Methyl-transfer activity measurement of purified Trm11-Trm112 complex. ^{3}H-methyl group incoorporation into $tRNA^{Phe}$ transcript was measured at 0, 1, 2, 4 and 8 min. The data is an average of three independent experiments.
